# Endoscopic features for early decision to evaluate superior mesenteric artery syndrome in children

**DOI:** 10.1186/s12887-021-02848-0

**Published:** 2021-09-08

**Authors:** Jae Young Kim, Myung Seok Shin, Sunho Lee

**Affiliations:** 1 Department of Pediatrics, Gyeongsang National University Changwon Hospital, 11 Samjunga-Ro, Sungsan-Gu, Changwon, 51472 South Korea; 2 Department of Pediatrics, Chungnam National University School of Medicine, Daejeon, South Korea; 3 Department of Pediatrics, College of Medicine, The Catholic University, St. Mary’s Hospital, Daejeon, South Korea

**Keywords:** Superior mesenteric artery syndrome, Children, Endoscopy, Duodenum

## Abstract

**Background:**

Diagnostic delay of superior mesenteric artery syndrome (SMAS) is common due to its rarity and lack of index of clinical suspicion. Early diagnosis under suspicion is pivotal for adequate treatment. Present study aims to explore the endoscopic features for early decision to evaluate SMAS in children.

**Methods:**

In case controlled observation study, the recruitment was limited to patients who had endoscopic finding I or finding 1 plus more as follows: a pulsating vertical or oblique band or slit like luminal narrowing of the third part of the duodenum without no expansion over one third during air insufflation for at least 15 s (finding I), a marked dilation of the duodenal first and second part during air insufflation at the third part of the duodenum (finding II), a bile mixed fluid collection (bile lake) in the stomach (finding III). SMAS was confirmed with UGI series or hypotonic duodenography in enrolled patients. We analyzed positive endoscopic findings related with SMAS.

**Results:**

The enrolled 29 patients consisted of 18 (62.1%) with SMAS and 11 (37.9%) without SMAS. The three most common presenting symptoms were abdominal pain, postprandial discomfort, and early satiety. The clinical impressions based on history and physical examination before endoscopy were functional dyspepsia (34.6%), gastritis or gastric ulcer (31.0%), and SMAS (17.3%). The constellation of three endoscopic findings (finding I + II + III, feature D) observed in 13 (72.2%) patients of SMAS group and 3 (27.3%) patients of non SMAS group (*P* = 0.027). Of 16 patients with features D, SMAS was diagnosed in 13 patients (81.2%) and not detected in 3 patients (18.8%) on UGI series or hypotonic duodenography.

**Conclusions:**

Endoscopic examination to the third part of the duodenum can provide a clue making a decision to evaluate SMAS, which consists of features of three endoscopic findings as follows: a pulsating vertical or oblique band or slit like luminal narrowing of the third part of the duodenum without no expansion over one third during air insufflation for at least 15 s, a marked dilation of the first and second part of the duodenum, and a bile lake in the stomach.

## Background

Superior mesenteric artery syndrome (SMAS) is a rare symptom complex condition caused by external compression of the third part of the duodenum between the aorta and the SMA [1].

A diagnosis of SMAS is challenging because of its rarity, nonspecific clinical presentations, and lack of high indices of suspicion [2, 3]. So, diagnostic delay is common and it is often diagnosed by incidentally identified external compression of the third part of the duodenum during investigative process of exclusion [2, 3].

As a consequence of diagnostic delay and following ineffective treatment, many patients suffer from upper gastrointestinal (UGI) upsets and related comorbidities such as food intolerance, undernutrition, weight loss, electrolyte imbalance and, poor quality of life [2–4]. Thus, early diagnosis under suspicion is essential to avoid these problems and carry out adequate treatment for recovery. SMAS can be confirmed radiologically. The UGI series or hypotonic duodenogaraphy remains mainstay for diagnosis. Contrast enhanced computed tomography (CECT), magnetic resonance angiography (MRA), or ultrasonography provide information on the angle and distance between the aorta and the SMA [3, 5, 6].

Most patients with SMAS present with vague and quite similar symptoms to common UGI disorders [1, 2, 4]. Therefore, most commonly undergo esophagogastroduodenoscopy (EGD) not UGI or hypotonic duodenography for initial investigative process. Till now, it has been known that EGD hardly affords an any information to make a suspicion or suggestion of SMAS. We hypothesized that endoscopic examination down to the third part of the duodenum may give a clue that reflect external compression of the third part of the duodenum in patient with SMAS. We performed our study to explore for endoscopic features related with external compression of the third part of the duodenum by the SMA through the analysis of endoscopic findings of patients who confirmed SMAS.

## Methods

### Patient selection, study design, and data analysis

**Fig. 1:**
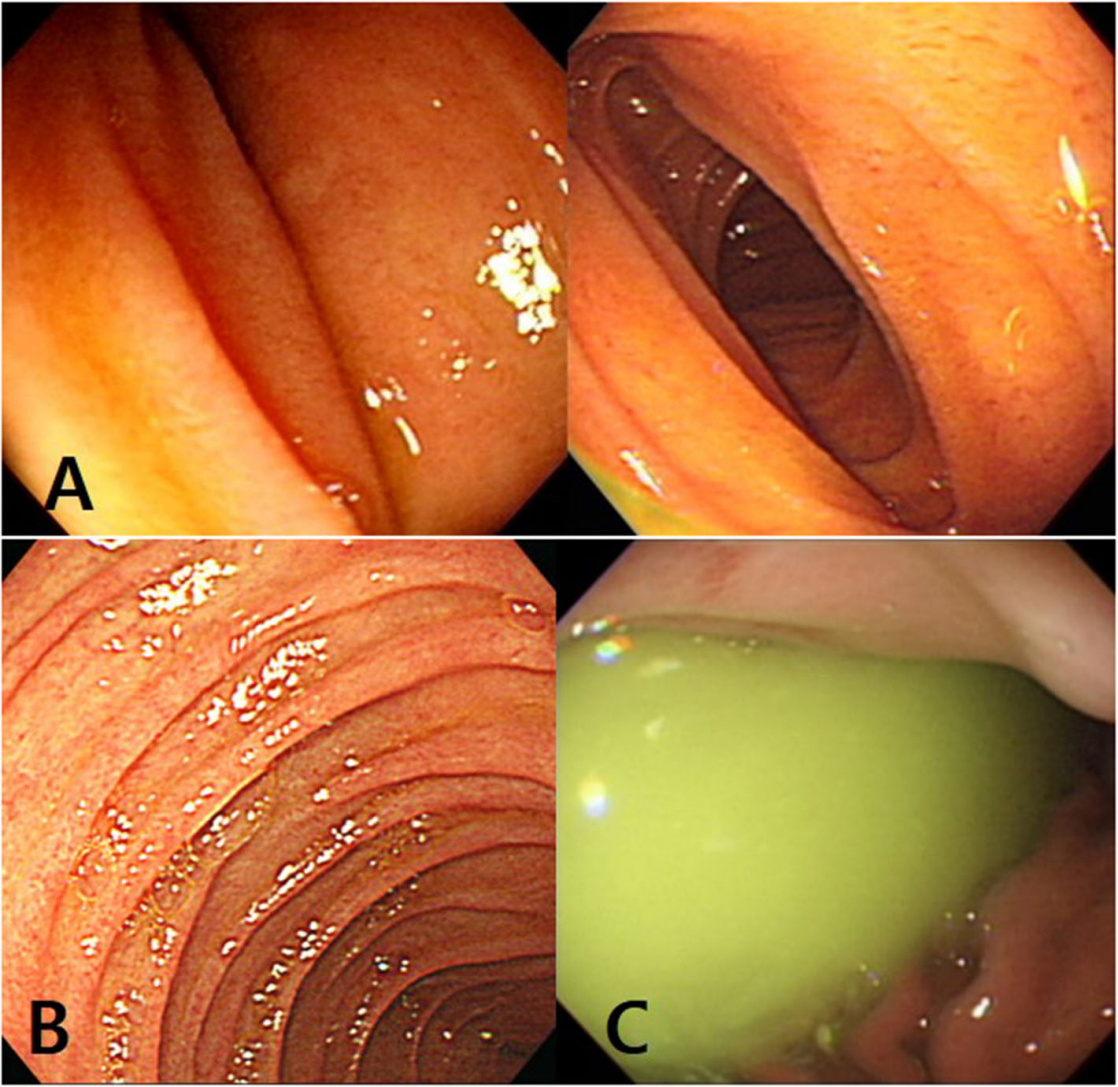
Photos of esophagogastroduodenoscopy. **a** A pulsatile band or slit like vertical or oblique band or slit like luminal narrowing and opening less than one-third of the third part of the duodenum with air insufflation over 15 s (finding I). **b** An over expansion of the first and second part of the duodenum during insufflation of the third part (finding II). **c** A large amount of bile mixed fluid (bile lake) in the stomach (finding III)

**Fig. 2:**
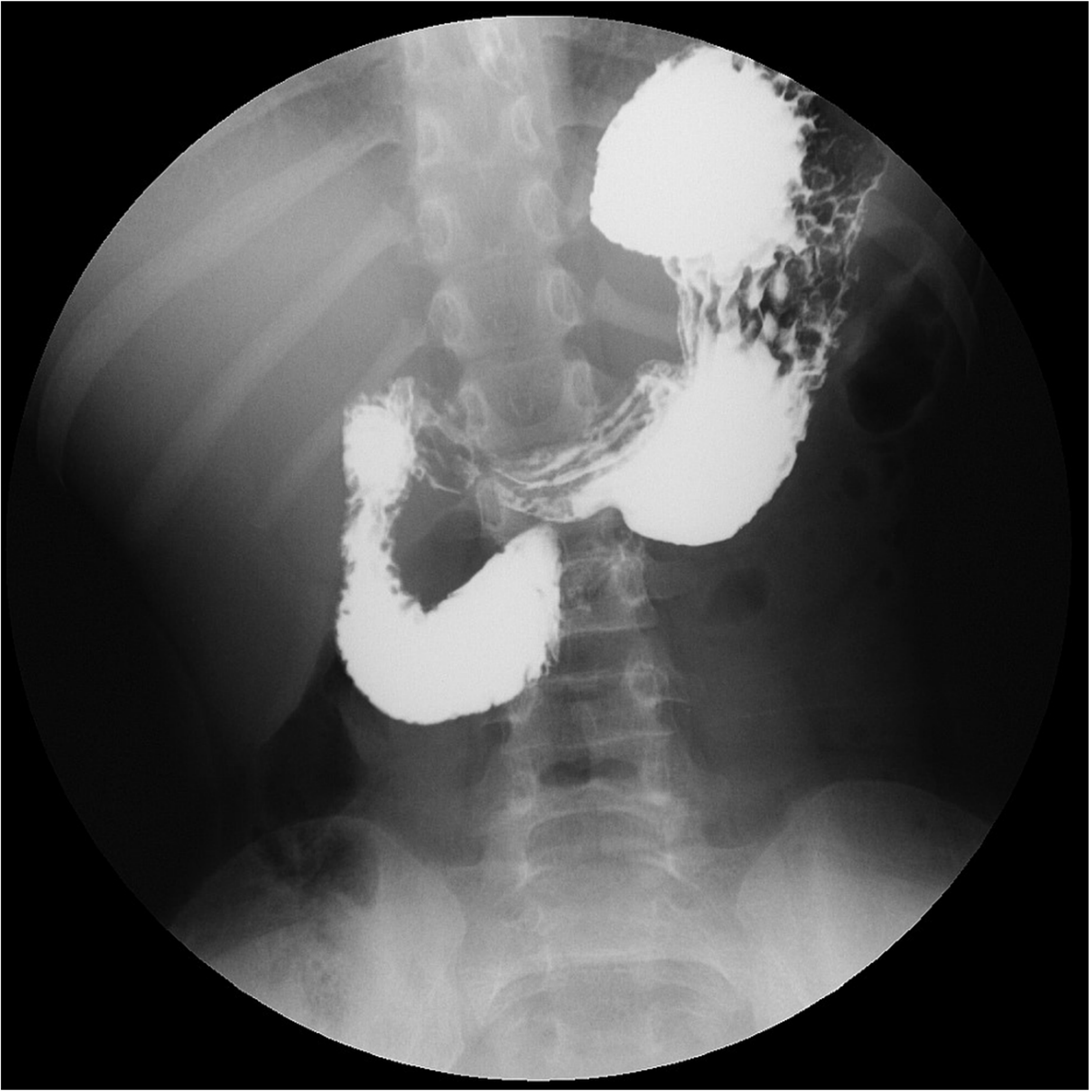
Upper gastrointestinal series shows abrupt vertical or oblique cutoff of contrast at the midline of the third lumbar level. The upper edge of the cutoff is sharp (white arrow), and lower edge is blunt and smooth (black arrow). These reflect the external compression of duodenum by the superior mesenteric artery

**Fig. 3:**
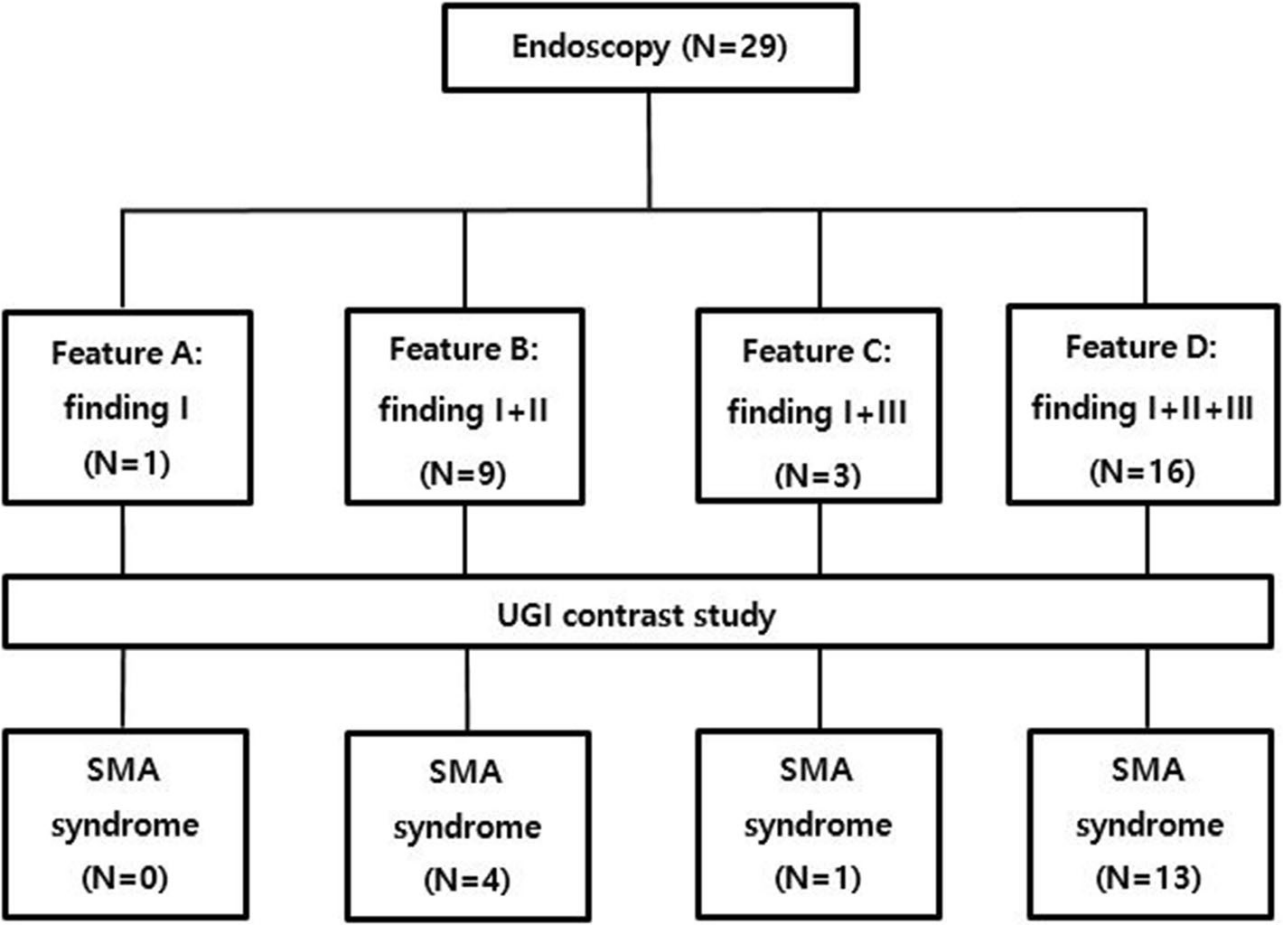
The Proportion of superior mesenteric artery syndrome according to the findings of endoscopy in the enrolled patients. Feature A, a pulsatile vertical or oblique band or slit like luminal narrowing with opening less than one third of the third part of the duodenum during air insufflation; Feature B, feature A plus a proximal duodenal dilation from the third part of the duodenum; Feature C, feature A plus a large amount of bile mixed fluid (bile lake) in the stomach; Feature D, feature B plus bile lake

We retrospectively or prospectively had collected data on patients who underwent EGD and UGI series or hypotonic duodenography with or without CECT scan due to UGI symptoms at the Department of Pediatrics, Chungnam National University Hospital since 2007. EGD was performed by the same pediatric gastroenterologist. We identified three endoscopic findings during EGD in some patients presented with UGI symptoms, which were as follows: a pulsating vertical or oblique band or slit like luminal narrowing with partial luminal opening less than one-third during air insufflation more than 15 s at the third part of the duodenum (finding I), with or without an over expansion of the second part of the duodenum during air insufflation at the third part of the duodenum (finding II), with or without a large amount of bile mixed fluid (bile lake) in the stomach (finding III) (Fig. 1). We classified endoscopic findings into 4 features as follows: feature A by finding I, feature B by finding I and II, feature C by finding I and III, and feature D by the constellation of finding I, II and III. The recruitment was limited to patients with one of the four endoscopic features. All enrolled cases underwent UGI series or hypotonic duodenography to confirm SMAS. A pediatric radiologist performed and interpreted the UGI series or hypotonic duodenography. In our study, the positive findings of UGI series or hypotonic duodenography for diagnosis of SMAS included as follows: (1) gastroduodenal dilatation with delayed gastric and duodenal emptying (2) abrupt vertical or oblique cutoff of contrast shadow at the third part of the duodenum (Fig. 2) (3) ‘to and fro’ flow of contrast from the proximal to the obstruction (4) with or without additional findings of slightly increase passage of contrast beyond the narrowing duodenal portion by positon change to prone [4, 5]. Figure 3 shows the evaluation flow of the enrolled cases and the results.

All enrolled cases were divided into 2 groups according to the results of UGI series or hypotonic duodenography. Group I included patients with SMAS and group II included control patients without SMAS. The medical records, including endoscopic findings, demographics, clinical presentations, growth status with weight, weight-for-age Z score, body mass index (BMI), BMI-for-age-Z score, and weight-for-height Z score were analyzed. The Z scores were assessed according to the stature percentiles of the Korean National Growth Charts drafted by the Korea Centers for Disease Control and Prevention.

### Statistical analysis

Statistical analysis on the data was performed by using IBM SPSS Statistics Version 24.0 (IBM Corp., Armonk, NY, USA). Demographics, duration of illness and growth status were assessed by the Mann-Whitney *U* test. Clinical presentations and endoscopic findings were assayed using the Fisher’s exact test. A value of *p* < 0.05 was considered significant.

## Results

The total number of cases available for the study was 29; group I included 18 cases with SMAS and group II included 11 cases without SMAS. The median ages of group I and II were 11.5 years (range, 7.8–16.2 years) and 13.2 years (range, 10.5–15.8 years) and male to female ratios were 0.8:1 in group I and 0.6:1 in group II, respectively. The median duration of symptoms before diagnosis was 68 days (range, 5–760 days) and 30 days (range, 5–1825 days) in each of the groups. The three most common presenting symptoms were abdominal pain (61.1% in group I and 90.9% in group II), postprandial discomfort (55.6% in group I and 54.5% in group II), and early satiety (50.0% in group I and 18.2% in group II). Weight loss was noted in 38.9 and 18.2% in group I and II, respectively. Less common symptoms included anorexia (33.3% in group I and 9.1% in group II), vomiting (27.8% in group I and 18.2% in group II), and nausea (16.7% in group I and 9.1% in group II). These presenting symptoms and signs were not statistically different between the groups (Table 1).

**Table 1:** Demographic Data and Clinical Features in Both Groups

Variable	Group I, *n* = 18	Group II, *n* = 11
Age (years)*	11.5 (7.8 ~ 16.2)	13.2 (10.5 ~ 15.8)
Female/male	10/8	7/4
Duration of symptoms (days)	68 (5–760)	30 (5–1825)
Presentation (%)		
Abdominal pain	11 (61.1)	10 (90.9)
Postprandial discomfort	10 (55.6)	6 (54.5)
Early satiety	9 (50.0)	2 (18.2)
Weight loss	7 (38.9)	2 (18.2)
Anorexia	6 (33.3)	1 (9.1)
Vomiting	5 (27.8)	2 (18.2)
Nausea	3 (16.7)	1 (9.1)

Results of age and duration of illness expressed as median (range)

Enrolled cases had more than one symptoms

*P = 0.041

Table 2 shows growth status including the mean body weight, weight-for-age Z score, body mass index (BMI), BMI-for-age Z score, and weight-for-height Z score. These parameters were not significantly different in both groups.

**Table 2:** Comparison of Growth Status in Both Groups, expressed as median and range

Variable	Group I (*n* = 18)	Group II (*n* = 11)
Weight, kg	36 (22 ~ 61)	45 (29 ~ 56)
Weight-for-age Z score	−0.68 (− 1.57 ~ 1.15)	−0.29 (−2.22 ~ 0.52)
BMI, kg/m^2^	15.9 (13.2 ~ 19.7)	18.2 (12.8 ~ 19.3)
BMI-for-age Z score	−1.08 (−2.08 ~ 0.14)	−0.55 (−3.16 ~ 0.27)
Weight-for-height Z score	−1.53 (−3.24 ~ 1.1)	−0.72 (− 3.29 ~ 2.53)

Statistically no significance between two groups

Table 3 shows the proportion of patients in the first clinical impression based on the history and physical examination, those with a constellation of three endoscopic findings, and findings consistent with SMAS in UGI or hypotonic duodenography. The three most common initial impressions before EGD were functional dyspepsia (34.6%), gastritis or gastric ulcer (31.0%), and SMAS (17.3%). Other impressions were reflux esophagitis (6.9%), functional abdominal pain (3.4%), small bowel obstruction (3.4%), and Crohn’s disease (3.4%). With UGI series or hypotonic duodenography, SMAS was diagnosed in 18 patients (62.1%) of the enrolled cases.

**Table 3:** Clinical impressions, EGD findings, and the results of UGI or hypotonic duodenography of 29 Patients

Variable	Impression(%)^a^	EGD^b^ (%)	UGI^c^ (%)
Functional dyspepsia	10 (34.6)	6 (20.7)	7 (24.1)
Gastritis or peptic ulcer	9 (31.0)	4 (13.8)	5 (17.2)
SMA syndrome	5 (17.3)	4 (13.8)	4 (138)
Reflux esophagitis	2 (6.9)	1 (3.5)	0 (0.0)
Functional abdominal pain	1 (3.4)	0 (0.0)	0 (0.0)
Small bowel obstruction	1 (3.4)	1 (3.5)	1 (3.5)
Crohn’s disease	1 (3.4)	0 (0.0)	1 (3.5)

^a^The first clinical impression based on the history and physical examination

^b^ A constellation of three endoscopic findings including a pulsating band or slit like luminal narrowing of the third part of the duodenum, a marked expansion of the first and second part of the duodenum during the third part insufflation, and a bile lake in the stomach

^c^ The finding consistent with SMA syndrome

EGD showed the following results: feature A in 1 case, feature B in 9 cases, feature C in 3 cases, and feature D in 16 cases. The cases of SMAS confirmed with UGI series or hypotonic duodenography according to each EGD feature were as follows: none (0%) in 1 case with feature A, four (44.4%) in 9 cases with feature B, one (33.3%) in 3 cases with feature C, and 13 (81.2%) of 16 cases with feature D (Fig. 3). The most common endoscopic finding associated with SMAS was feature D, which was documented in 13 (72.2%) patients in group I and 3 (27.3%) patients in group II, respectively (*P* = 0.027) (Table 4). Of 16 patients with features D, SMAS was diagnosed in 13 patients (81.2%) and not detected in 3 patients (18.8%) on UGI series or hypotonic duodenography. There were no statistically significant in feature A, B, and C between both groups.

**Table 4:** Comparison of Endoscopic Findings in Both Groups

Variable	Group I, *n* = 18 (%)	Group II, *n* = 11 (%)	*P* value
Feature A (Finding I)	0 (0.0)	1 (9.1)	0.379
Feature B (Finding I + II)	4 (22.2)	5 (45.4)	0.114
Feature C (Finding I + III)	1 (5.6)	2 (18.2)	0.539
Feature D (Finding I + II + III)	13 (72.2)	3 (27.3)	0.027

Fining I, a vertical or oblique pulsatile band or slit like luminal narrowing and opening less than one third of the third part of the duodenum with air insufflation over 15 s; Finding II, an over expansion of the first and second part of the duodenum during air insufflation of the third part; Finding III, a large amount of bile mixed fluid (bile lake) in the stomach

## Discussion

The aim of the present study was to explore whether the EGD provides clue on early decision to perform a diagnostic test for SMAS. To the best of our knowledge, no study on the endoscopic features related to SMAS has yet been conducted. Our study suggests that endoscopy down to the third part of the duodenum can give a significant information in deciding to perform a diagnostic test for SMAS. The endoscopic clue in our study was a constellation of three findings including pulsating vertical or oblique band or slit like luminal narrowing of the third part of the duodenum with luminal expansion no more than one third during air insufflation over 15 s, proximal duodenal over distension during air insufflation in the third part of the duodenum, and bile lakes in the stomach.

SMA syndrome is a symptom complex disease caused by extrinsic compression of the third part of the duodenum due to narrowing of the aortomesenteric space (AMS) between the aorta and the SMA [1]. Although the origin and route of the SMA are variable, it arises classically from the aorta at the level of intervertebral discs between vertebral level L1 and L2 [7]. The third part of the duodenum extends from the right side of L3 or L4 to the left side of the aorta and runs horizontally at the level of L3 [8, 9]. Therefore, narrowing of aortomesenteric angle (AMA) and shortening of aortomesenteric distance (AMD) can compress the third part of the duodenum. Risk factors related to narrowing of AMA include high fixation of the duodenum by abnormally high insertion of the ligament of Treitz, abnormally low origin of the SMA, incomplete rotation of the duodenum, anomalies of the SMA, rapid linear growth with insufficient weight gain, rapid weight loss, and surgery for scoliosis, [1, 9–11]. SMAS often develops in association with these risk factors but, up to 40% cases of SMAS occur without being related to these risk factors [12]. Weight loss and low BMI are often reported in cases with SMAS, which can be not only clinical manifestations of SMAS, but also a consequence of poor oral intake due to symptoms related to SMAS [2, 4, 13]. Weight loss and low BMI are not always identified in pediatric population with SMAS [2, 13]. In our study, no substantial differences were observed in number of cases of weight loss, BMI, and weight-for-height Z score between both groups (Tables 1, 2).

Since SMAS rarely occurs and present nonspecific UGI symptoms with lack of clinical suspicions, making a diagnosis with early suspicion is a challenge. So, a diagnosis of SMAS may commonly be delayed for a period of time. The median duration of symptoms before diagnosis varies from 5 to 30 days and up to 18 months (range 0–900 days) according to the literature [4, 14, 15]. In our study, the median time interval between symptom onset and diagnosis was 68 days (range 5–760 days).

Many clinicians remain unaware of this syndrome, which may often be diagnosed during the investigative process of excluding other suspected conditions [3, 13]. The diagnosis of SMAS is done radiologically by demonstrating an evidence of external compression of the third part of the duodenum. These can be documented by UGI series, hypotonic duodenography, CT, or MRA. In the literature, most studies used UGI series or CT in diagnosis of SMAS. The compatible findings of UGI series or hypotonic duodenogaphy, which is a mainstay in diagnosing SMAS, includes an abrupt vertical or oblique cutoff of contrast just right of midline at the third part of the duodenum with dilation of the first and second parts of the duodenum, delayed gastric and duodenal emptying, and to and fro pattern of antiperistaltic waves proximal to the third part [1, 3, 6]. In addition, patients with true vascular compression of the duodenum often shows increase of passage of contrast to the distal duodenum by repositioning into a left lateral or a prone position [3, 6].

Despite the diagnostic criteria in UGI series, Levin disputed many of the UGI pictures cited in the literature used to diagnose SMAS. Levin determined on analyzing CT and MRI that the AMS is located along the L3 midline or with a slight deviation of the SMA to the right in most cases and less often to the left. The diameters of the aorta and the SMA at the level of L3 are 2 cm and 0.5 cm, respectively. Thus, the length of the narrowed part of the duodenum between the aorta and the SMA can’t exceed 1 cm [16]. On the radiometric analysis of X-rays in 35 articles devoted to SMAS, only 6 (17%) of 35 cases showed that narrowed part of the duodenum was located within 1 cm length between the aorta and the SMA [16]. In the remaining 29 cases, the beginning of the narrow segment was 2.5–4.6 (3.2 ± 0.15) cm proximal to the SMA. Levin argued that the length and location of the narrowing of the duodenum do not match with the location of the AMA [16, 17]. Although the anatomic variation and shape of the SMA are quite diverse, Levin predicated that the closed segment of the duodenal images in 29 cases seem to be unlikely related to the SMA through the radiometric analysis from the 35 published articles [7, 13, 16]. Based on these analysis, Levin introduced the theory of Ochsner functional sphincter dyskinesia as a cause of manifestations of SMAS. Levin assert that Ochsner sphincter normally contracts in response to the penetration of the acidic gastric contents into the duodenum and prevents the penetration of chime with a low pH into the jejunum [16].

Levin stated that Ochsner sphincter can be seen through an oral contrast study using 200 ml of barium with 3 g of vitamin C added. However, the clinical evidence of Ochsner sphincter has not been studied and certified by other researchers to assert as an indisputable scientific fact so far. The theory of Ochsner functional sphincter dyskinesia has limitations in explaining the followings. In our study, all the patients with SMAS had a bile lake, which alkalinizes the gastric content, and many patients had taken proton pump inhibitors (PPIs) or histamine 2 blockers before diagnosing SMAS, so the passage of strong acid chime rarely occurs into the duodenum. In the study of Ganss et al., all the operated patients received PPIs for years before surgical treatment and so the acidity decreased in their stomach and duodenum [13]. In our study, endoscopic findings suggest that the pulsating oblique or vertical band or slit like luminal narrowing reflect external compression rather than functional sphincter contraction (Fig. 1). Nevertheless, Levin’s radiometry analytic study raised that diagnosing SMAS without considering the pathological physiology based on the anatomy of the disease may lead to an inappropriate or suboptimal treatment. Further studies are needed to address Levin’s assertion. We would propose to perform combination tests of UGI series and ultrasonography. When a duodenal cutoff is observed in UGI or hypotonic duodenography, simultaneous performed ultrasonography can clarify whether the duodenal cutoff of contrast is formed by the SMA or not. Recently, we are trying to perform combination tests of UGI series with using 3 g Vitamin C mixed 200 ml barium and simultaneous ultrasonography in patient with endoscopic feature D.

In the literature, CECT, MRA, or ultrasonography have been used to measure the anatomical state of the AMA and AMD supporting the diagnosis of SMAS [13, 18, 19]. An AMA < 22–25° and AMD < 8 mm correlated well with the development of symptoms of SMAS in adults [6, 18–20]. And these are consequently used as cutoff values for diagnosis of SMA syndrome in adults. However, there are cases that have no SMAS even if they have sufficient cutoff values, and normal ranges of cutoff values are variable in pediatric population [14, 21]. Desai et al. observed a strong positive correlation between BMI and the AMA, and less chance of developing SMAS with increment in BMI [22]. SMAS had not documented in 25% patient with these rates in a prospective study of 100 patients who had undergone CT scan for various other complaints [22]. Therefore, when abdominal CT or MRA is used to diagnose SMAS, it should be interpreted with caution, especially in the pediatric population. Sinagra et al. stated that AMD seems to be more accurate rather than AMA to diagnose SMAS because the anatomy of SMA is variable and there is no an agreement how AMA should be measured radiologically [23].

Although we suggested the argues on the diagnostic test of SMAS, early diagnosis under suspicious clues is important to avoid unwanted problems and achieve rapid recovery. Most patients with SMAS may suffer from UGI symptoms and related comorbidity such as poor oral intake, weight loss, undernutrition, electrolyte abnormalities, and poor quality of life [2, 4, 12–14]. So, many patients with SMAS were received EGD because of common UGI symptoms. EGD is useful to differentiate the UGI diseases such as esophagitis, gastritis, *H. pylori* infection, bile reflux, or duodenitis but, it is not possible to make a diagnosis of SMAS. Lippl et al. suggested that endoscopic findings such as duodenal dilatation, liquid stasis, and antiperistaltic waves may suggest SMAS [24]. Meanwhile, Sundaram et al. stated that although the Lippl’s suggested endoscopic findings may suggest duodenal obstruction, a diagnosis of SMAS cannot always be made with certainty with those findings [25]. They recommended using both endoscopic ultrasound and endoscopy for the diagnosis of SMAS [25]. Unlike this, Sinagra et al. suggested that documentation of pulsatile extrinsic compression in the third part of the duodenum by EGD is the most reliable finding to suspect SMAS [23]. Cappell et al. reported a case of SMAS with an endoscopic photograph showing pulsatile band like luminal narrowing of the third part of the duodenum which was gradually, partially opened by moderate air insufflation [26]. We observed that diagnostic yield of SMAS was significantly higher in patients with endoscopic feature D compared to those without that. The sensitivity and the positive predictive value having SMA syndrome in cases with endoscopic type D were 72.2 and 81.3%, respectively. Therefore, we suggest that endoscopic examination down to the third part of the duodenum can provide a meaningful clue in deciding to investigate for SMAS.

## Conclusion

In conclusion, clinical symptomatology, physical examination, and endoscopic information are important for early suspicion of SMAS. Even though our study has some limitations due to the small number of enrolled patients and lack of endoscopic follow-up after improvement of symptoms, we recommend to examine down to the third part of the duodenum when thick bile stained fluid retention (bile lake) is noted in the stomach during EGD in patient with postprandial distress, early satiety, anorexia with or without weight loss. We suggest an endoscopic feature D would be a substantial clue to reach an early suspicion and make a decision for evaluation of SMAs. Further clarification with future large scale case studies are warranted.

## Data Availability

The datasets generated and analyzed during the present study are available from the corresponding author on reasonable request.

## Abbreviations

SMA: Superior mesenteric artery
GI: Gastrointestinal
CECT: Contrast enhanced computed tomography
MRA: Magnetic resonance angiography
AMD: Aortomesenteric distance
AMA: Aortomesenteric angle
EGD: Esophagogastroduodenoscopy
